# Heidelberg Retina Tomography Analysis in Optic Disks with Anatomic Particularities

**Published:** 2010-11-25

**Authors:** AM Dascalu, C Alexandrescu, R Pascu, R Ilinca, V Popescu, R Ciuluvica, L Voinea, C Celea

**Affiliations:** ** ‘Carol Davila’ University of Medicine and Pharmacy, BucharestRomania; ** Clinica Oftalmologie, Spitalul Universitar de Urgenta , BucharestRomania; *** Agrippa Ionescu Hospital, BucharestFrance

**Keywords:** HRT, Glaucoma, megalopapilla, microdisc

## Abstract

Due to its objectivity, reproducibility and predictive value confirmed by many large scale statistical clinical studies, Heidelberg Retina Tomography has become one of the most used computerized image analysis of the optic disc in glaucoma. It has been signaled, though, that the diagnostic value of Moorfieds Regression Analyses and Glaucoma Probability Score decreases when analyzing optic discs with extreme sizes. The number of false positive results increases in cases of megalopapilllae and the number of false negative results increases in cases of small size optic discs. The present paper is a review of the aspects one should take into account when analyzing a HRT result of an optic disc with anatomic particularities.

One of the most used clinical and imagistic argument for glaucoma is increased cup/disc ratio. It is generally accepted that normal subjects have a small cup, with c/d ratio of 0.1–0.3 and that a large cupping, with a c/d ratio of 0.7–0.8 is characteristic for glaucoma. However, all systems of diagnosis based on c/d ratio, clinical or imagistic, suffer some limitations, especially in case of an optic disc with anatomic particularities. One of the most frequently encountered problems is that the disc area influences significantly the cup area. In a study about the diagnostic accuracy of HRT and GDx– 2 of the most used imaging techniques used in glaucoma–,Mladin and col. observed that an increased number of healthy subjects with large disc area were considered as glaucomatous by computerized analysis. The implications are clear and clinically relevant:  HRT and GDx, as an extension of C/D ratio system of diagnosis, are not capable of identifying glaucomatous subjects by those with megalopapilla. A big cup does not always mean glaucoma. Not presented in the study, but of equal importance is the case of subjects with microdiscs. In these cases, the glaucomatous damage may be ignored until advanced stages.

The present article is a review of some aspects to take into account when analyzing a HRT result in an optic disc with a particular structure:

## Megalopapilla

Megalopapilla is defined as an optic disc area of 2.5 mm^2^ (or 2.85 mm^2^–Conley E.J. , Optic Disc Area Asymmetry May Also Play a Significant Role in Glaucoma When Evaluating Patients With Macro–Disc Optic Nerves), with increased cupping, but normal intraocular pressure and normal visual field. 

HRT particular findings:

- round cup, not characteristic for glaucoma
- increased rim area and rim volume (at the superior normal limit or more)
- increased linear and area C/D ratio
- larger circumference of the megalopapillae leading to a horizontally stretched contour line, which, therefore seems flatter compared to a contour line of a normal disc;
- mean RNFL thickness may be slightly reduced;
- cup shape measure (CSM) may be outside normal limits
- height variation contour may be normal or increased
- high level of the reference plan (red line)

Moorfields Regression Analysis may find some sectors ‘outside normal limits’, especially the nasal ones, but if the global value is ‘normal’, this finding may be often clinically irrelevant.

Leaving brightness control to the automatic mode, megalopapillae tend to be underexposed, because of intense reflection of the large cup area. The optic disc contour is, thus, not always easy to draw. These difficulties may also appear when using the interactive mode. Horizontal and vertical retinal profiles may reveal ‘steps’ of the temporal peripapillary retina; if they are included in the contour area, the optic disc may be diagnosed as glaucomatous by the automated software. Three–dimensional reconstruction of the optic disc may be useful in those cases for a most accurate drawing of the optic disc contour line.

**Figure 1:**
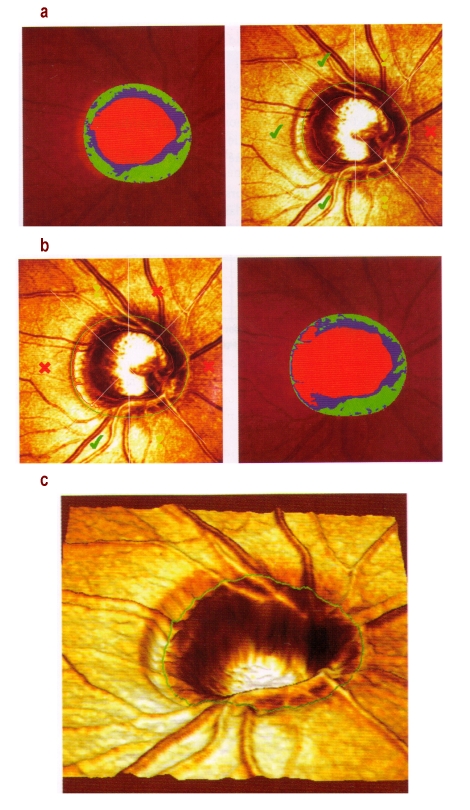
a) megalopapilla; correct contour line placement, at the scleral ring; b) incorrect placement of the contour including temporal ‘steps’ gives a glaucomatous aspect to the optic disc c) three-dimensional reconstruction helps correct identification of the optic disc contour (A.F. Scheuerle, E.Schmidt: Atlas of Laser  Scanning Ophthalmoscopy, Ed Springer 2004)

**Figure 2:**
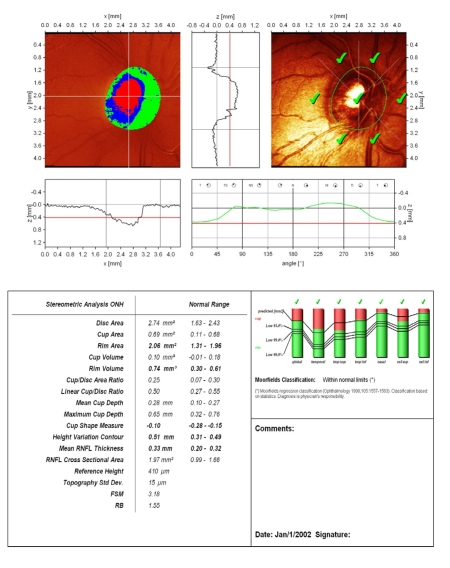
Normal aspect of Megalopapilla : disc area, rim area and rim volume are increased; ‘step’ of the temporal peripapillary retina, with correct placement of the contour line; large excavation with vertically pronounced oval shape; cup shape measure outside normal limits; increased height variation contour; both polar peaks(‘double humps’) reach the mean retina height; Moorfields Regression Analysis (MRA) is normal.

An accurate placement of the contour line is essential for the relevance of the results. We present a case of complete lack of concordance between MRA and GPS analysis, the cause being the failure of automated identification of the optic disc border in a patient with megalopapilla (disc area: RE 3.32 mm^2^; LE: 2.75 mm^2^). GPS is displaced, the signs are placed within optic disc area and the result is ‘outside normal limits’. Manually drawn optic disc border in MRA places the results within normal limits. 

**Figure 3:**
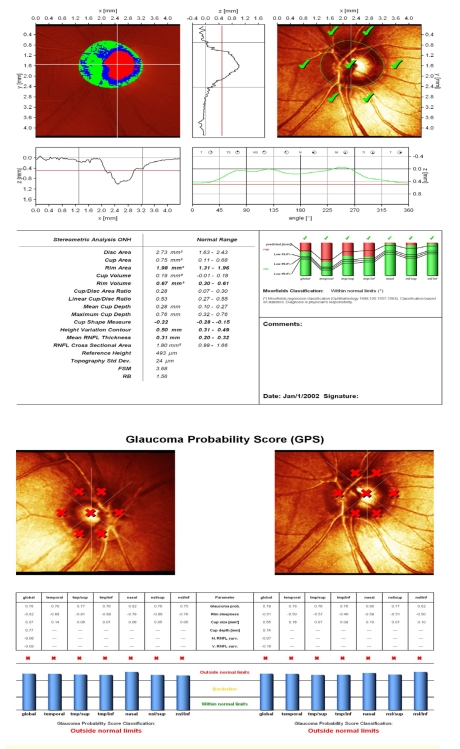


Reduced values of rim area and rim volume towards the inferior normal limits (early sign) or beyond these limits (in advanced glaucoma), flattened and asymmetric contour line, with the 2 polar peaks not reaching the mean retina height (red line), loosing the aspect of ‘double humps’ and finally, the change between 2 examinations of clinical significance (decrease of rim area, rim volume, mean RNFL thickness)–all of them are signs that must have a superior clinical value to automated analysis of the optic disc (MRA and GPS which compare the results to a statistical data–base), and, therefore, should be searched for .

**Figure 4:**
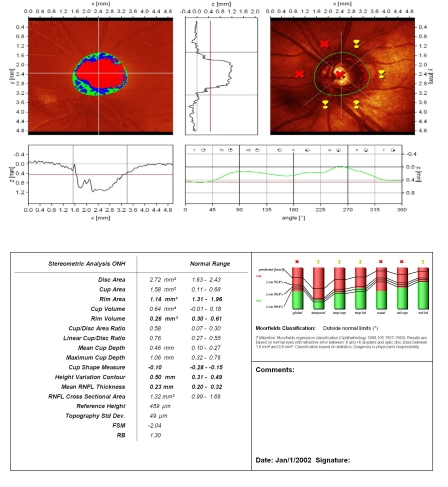
Advanced glaucoma and megalopapilla: increased disc area, but with rim area and volume and mean RNFL thickness below normal limits; superior ‘notching’ of the neuroretinal rim in temporal/superior sector; the presence of the vascular trunk gives a false increased rim area value in temporal/inferior sector; flattened and asymmetric height variation contour; superior ‘hump’ does not reach the mean retina height; MRA: outside normal limits;

Hawker and col. (Ophthalmology, 2006;113:778–785: Specificity of HRT's diagnostic algorithms in a normal elderly ) find that the sensitivity of HRT analysis decreases significantly with increased optic disc area.  Due to increased cup area, reduced RNFL thickness and reduced rim area, especially in the temporal and inferior sectors, large optic discs may be often classified as glaucomatous. A normative database for megalopapillae and the integration of disc area in a multivariate analysis may increase the diagnosis capacity of automated morphometric analysis (Mladin and col., Klinische Monatsblatter fur Augenheilkunde, 2006: 223:308–314). The authors conclude that:

- disc area must be taken into account in all cases with increased excavation suggestive of glaucoma
- it is better to use correction factors whenever analyzing optic discs of extreme sizes, including the examination by HRT or GDx
- Classifying Systems which include disc size (DDLS–disc damage likelihood scale, for instance) may represent an advantage in these cases

## Microdisc. Tilted disc

A disc area below 1.6 mm^2^ may also cause difficulties of diagnosis, especially in those cases associated with tilted disc. There are some aspects in HRT examination to take into account:

- Shallow excavation, in some cases almost absent;
- height variation contour: normal value;
- low–standard reference plan (red line), more important in cases of tilted discs;
- dynamic and symmetric contour, far above the mean retina height;
- in cases with tilted disc, temporal region, often the one used for calculation of the reference plane, is significantly lower than the nasal sector;
- The automated interpretation of data is thus applicable only to the temporal part, but, in fact, it is applied to the entire disc area. Due to these differences of height between temporal and nasal part, the results may be ‘within normal limits’ , with false increased values of rim area and rim volume, until advanced glaucomatous damages occur;
- the cup appears shallow, small, with a vertically pronounced oval shape;

**Figure 5:**
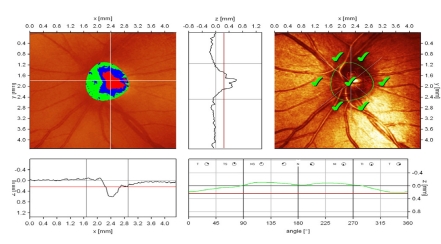
Small–size optic disc, with very small, shallow excavation, slightly eccentric because of the vascular trunk; rim area and rim volume are to the inferior normal limit; CSM and height variation contour are normal; both polar peaks (‘double humps’) are above the mean retina height (black line); low standard reference plane (red line); vital appearance of the peripapillary retinal nerve fiber layer with good reflectivity; Moorfields Regression Analysis: ‘within normal limits’

**Figure 6:**
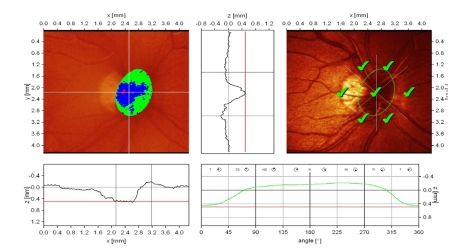
Tilted small–size optic disc, with shallow excavation; temporal peripapillary atrophy; the optic disc contour is difficult to define; height variation contour is above mean retina height (black line);  asymmetry with difference in height between temporal and nasal sectors of the optic disc;

**Figure 7:**
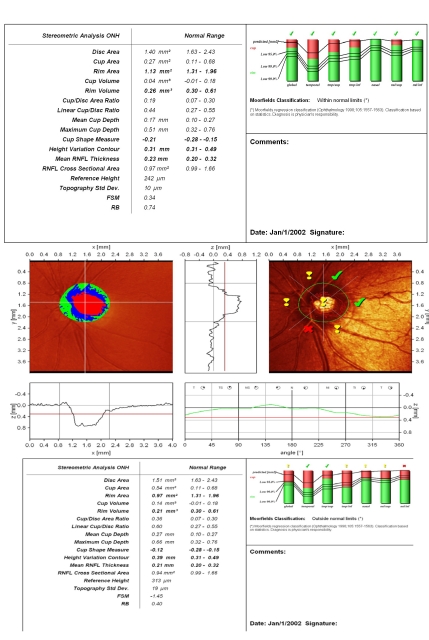
Small disc, with decreased values for rim area and rim volume; CSM outside normal limits; flattened height variation contour, without the physiologic ‘double humps’ appearance; false increase of rim area and rim volume of the nasal and nasal/superior sectors due to the prominence of the vascular trunk; linear C/D ratio and C/D area ratio are slightly increased, but within normal limits; ‘notching’ in the temp/inferior sector; Moorfields Regression Analysis: outside normal limits

## Optic Disc Drusen

HRT characteristic aspects:

- prominent optic disc, with no excavation;
- the contour line follows the drusen area, which oversize optic disc margins;
- some drusens can be differentiated based upon the relatively increased intensity of the signal resulting from their characteristic shape at the surface of the optic disc;
- horizontal and vertical profiles show the prominence of the drusen;
- variable form of the contour line height profile;

It is easy to see that the analysis of the key elements for glaucoma (excavation, C/D ratio, neuroretinal rim) is significantly affected by the presence of the drusen. In these cases, the changes in time are the most clear diagnostic element in glaucoma suspects.

**Figure 8:**
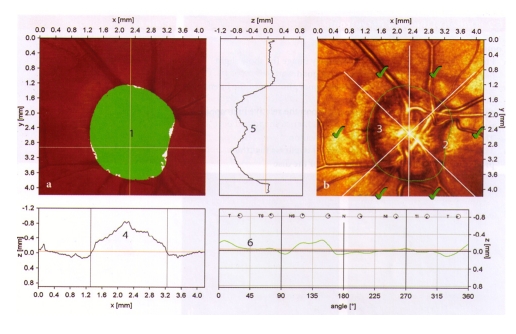
Prominent drusen in vertical adn horizontal profiles, with no excavation; the stereometric parameters (rim area, rim volume, C/D ratio) are completely irrelevant.

## HRT in myopic patients 

Clinical studies revealed progressive thinning of the RNFL thickness in imagistic investigations of the optic disc with increasing of myopia. More constantly, the changes appear in the 120 degrees superior region of the papilla (Kremmer and col., Arch Clin Ophthalmol 2004; 242: 489). Knowing the ‘normal aspect’ in these cases is important for the correct understanding of the results. 

Megalopapilla and peripapillary atrophy are also common findings in myopic patients, with all their characteristics above mentioned. 

Other considerations to take into account:

- the disc area varies with antero–posterior length of the eye: for each 1 mm, disc area increases with 0.065 mm^2^;
- with regard to the relation between refractometry and disc area, different studies showed different results: some reveal significant correlation between disc area and the absolute value of myopia (Jonas and Wang, Rotterdam Study), other find that between –8D and ±4D, there are no statistical significant correlation between ametropy and disc area (Optic Disc Measurements in Myopia with Optical Coherence Tomography and Confocal Scanning Laser Ophthalmoscopy; Christopher Kai–shun Leung, si col.)

HRT 3 software allows automated correction of the results with ametropy and keratometry (in GDx examination, a manual correction is needed).

## Amblyopia

Amblyopia is the clinical expression of a variety of ocular disorders. All of them have in common the lack of or insufficient development of nervous cortical connections, with secondary decreased visual acuity in absence of a local ocular cause. There are a few studies about HRT aspect of the optic disc in amblyopic eyes. It seems that HRT comparative examination of the optic discs can demonstrate the presence of amblyopia in children with strabismus or uncorrected anisometropy. (Optic Nerve Head Topographic Analysis and Retinal Nerve Fiber Layer Thickness in Strabismic and Anisometropic Amblyopia– Yassar Duranoglu and col., Annals of Ophthalmology, Sep. 2007). 

The results of HRT are difficult to classify as normal or glaucomatous, due to sector or global decreased values of rim area, rim volume or RNFL thickness, often associated with amblyopia.

**Figure 9:**
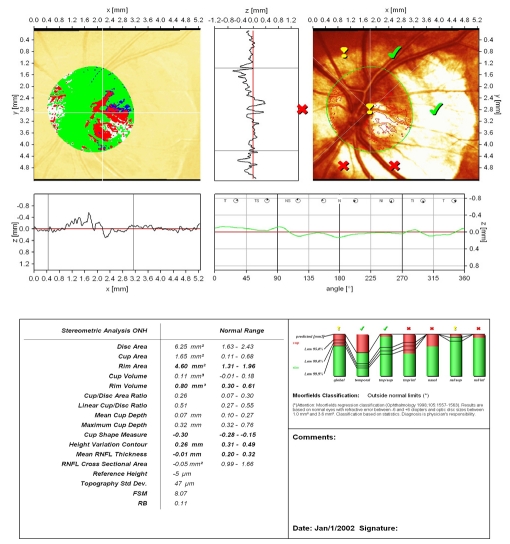
Megalopapilla and amblyopia: increased value of rim area and disc area, but with relatively decreased rim volume and RNFL thickness; clinical context: anisometropic patient with severe myopia (–18 D) in the left eye and consecutive severe amblyopia

## Age

Clinical studies (Kamal DS, Garway–Heath DF, Hitchings RA, Fitzke FW: Use of sequential Heidelberg retina tomograph images to identify changes at the optic disc in ocular hypertensive patients at risk of developing glaucoma. Br J Ophthalmol 2000;84:993–998, N Stroditis and col.,Factors affecting the test–retest variability of Heidelberg retina tomograph and Heidelberg retina tomograph 2  measurements, 2005) revealed a slight decrease of RNFL thickness with age. This observation should also be taken into account in clinical practice. 

## Conclusions

Automated analysis of the optic disc by Moorfields Regression Analysis (MRA) or Glaucoma Probability Score (GPS), which compare the results with statistic significant database, is not always clinically relevant in cases of optic discs with anatomic particularities. 

Rigorous observation of all HRT diagnosis algorithms (stereometric parameters, horizontal and vertical profiles, dynamic and symmetry of height variation contour, discriminant analysis, MRA and GPS) is extremely important.

HRT cannot replace a complete anamnesis and ophthalmologic exam as lack of understanding the clinical context can generate errors of interpretation (severe myopia, optic disc drusen, amblyopia).

In suspect optic discs, long term follow–up and detection of changes in the key–structures of the optic disc (cup, neuroretinal rim and RNFL) is the best argument for glaucoma diagnosis.
